# Treatment of Compound Fractures1Read before the Buffalo Academy of Medicine.

**Published:** 1896-02

**Authors:** Eugene A. Smith

**Affiliations:** Professor of general and descriptive anatomy, Niagara University, Buffalo, N. Y.; 771 Ellicott Street


### BUFFALO MEDICAL JOURNAL.

Vol. XXXV. FEBRUARY, 1896. No. 7.

### Original Communications.

### TREATMENT OF COMPOUND FRACTURES.[1]

By EUGENE A. SMITH, M. D.,
Professor of general and descriptive anatomy, Niagara University, Buffalo, N. Y.

IT IS sometimes reproachfully said that modern surgery leans too far toward radicalism, and that the good results obtainable by antiseptic and aseptic methods foster this tendency. If this be so, it is no less true that there is some conservatism in modern surgery, and this is nowhere more strikingly shown than in the treatment of compound fractures. It is also true that antiseptic wound treatment originated and justified this conservatism. In the old days, conservative surgery in the treatment of compound fractures resulted so disastrously that the accepted rule of practice was amputation for compound fractures of the long bones of the extremities ; and open treatment of the wound with drainage in all cases where amputation was impracticable. Contrasted with this radicalism of the old school of surgeons, mutilation by amputation is now the exception rather than the rule, and suppuration is less frequent, less prolonged and less often followed by fatal septicemia and pyemia.

To those who may be somewhat sceptical as to the truth of these generalisations, the following report of cases and observations based thereon may be of interest.

Compound fractures belong to the class of grave surgical conditions. They are the result of unusual violence and the various complications arising after simple fractures, such as shock, fat embolism, thrombosis, injury to vessels and nerves, osteomyelitis, necrosis, failure of union and intercurrent diseases are likely to occur more frequently and with more gravity. But the most dangerous factor in compound fractures is the element of septic infection. Sepsis jeopardises the welfare of the patient now in the

1. Read before the Buffalo Academy of Medicine.

same way that it did before the days of Lister and Pasteur. When infection once occurs these cases pursue a more or less prolonged course of suppuration and fever, ending in recovery only after a slow and precarious convalescence, or in progressive emaciation, deepening hectic fever, profound septicemia, pyemia and death. Old hospital reports show a mortality of from 50 to 75 per cent. after compound fractures, from septicemia and pyemia. One writer was able to report the unusual success of 48 per cent. of recoveries after conservative treatment of compound fractures. Bacteriological knowledge enables the modern surgeon to guard against the occurrence of infection in many cases and to cope more successfully with it when present. Recent statistics of 500 cases of traumatic compound fractures and designed osteotomies in hospital practice gave less than 1 per cent. of deaths from complications induced by septic infection. The statistics of traumatic cases alone are much less favorable, but far better than those already quoted of pre-antiseptic days.

Fractures are compound when in addition to the broken bone the soft parts are crushed, cut or torn, allowing communication between the site of the fracture and the air. Formerly the air was regarded as the source of infection. We no longer fear the entry of air as much as we do the contamination of the wound by contact with unclean objects. Contact infection is the main source of entry of pathogenic bacteria, notably the streptococci and staphylococci of pus.

Foreign bodies of the most diverse kinds, most diversely soiled and infected, may touch the wounded tissues after compound fracture, or be found in the wound itself. Shreds of cloth, particles of dirt, cinders, splinters of wood, bullets and substances designed to be hemostatic by well-meaning but surgically dirty friends, such as tobacco, cobwebs or rags, may lie openly in the wound or be hidden away in its nooks and crannies. Pyogenic bacteria, happily excluded in every other way, may be carried into the wound by the inverted, broken, unclean skin edges of the wound itself. Or, if by rare chance infection does not accidentally occur, the careless diagnostician, satisfying the vulgar curiosity of bystanders as well as his own, by inserting an unwashed finger or probe into the wound, deliberately jeopardises the patient's life, by planting germs in the most favorable soil for their development.

Autoinfection of a compound fracture not infected from without, or of a simple fracture with pus formation, and the production of a compound fracture by its evacuation, is a rare occurrence, but possible. In 1890, a tugboat fireman was brought to Mynter's clinic, suffering from a simple fracture of the tibia in its upper third and scalds of the second degree of the arms, neck and head, the result of a boiler explosion. Suppuration of the scalded surface was free. The fracture did well for a time. High temperature and chills, with local inflammatory signs, called attention to the fracture about the seventh day, and thigh amputation became necessary after the tenth day to overcome the acute septicemia, developed from suppuration at the site of fracture.

It may now be said that the question of treatment of compound fractures hinges mainly on the prevention and elimination of infection. If we succeed in preventing infection of the injured parts, the lapse of a few days, or of ten days at the most, converts the compound fracture into a simple fracture, which is treated as such during bony regeneration. If infection occurs and we wash away or destroy the bacteria before they can penetrate the tissues, we again convert the compound fracture into a simple one ; and lastly, if infection progresses beyond our reach, we must contend with the septicemia which supervenes. In all cases, therefore, it should be the rule of practice to convert a compound fracture into a simple one by excluding pathogenic bacteria from the wound or by removing and destroying them if they have gained entrance. Such treatment is exactly analogous to the aseptic and antiseptic treatment of incised wounds of the soft parts, and the result may be called healing of compound fracture by first intention.

Compound fracture occurs by direct violence when the force injures the soft parts and breaks the bone directly, as by the crush of a wagon wheel or the passage of a rifle ball. Indirectly, after force has broken a bone, a fragment, or the splintered end of the shaft may pierce the soft parts and skin. According to the degree of violence, the case may show moderate laceration of the skin with sharply cut edges, moderate contusion and tearing of the muscular and connective tissues, injury to small vessels and nerves, and one of the classical displacements of the bone ends, transverse, longitudinal, oblique or dentate ; or the violence being more severe, the condition may pass from severe laceration and contusion of the soft parts with comminution of bone and grotesque deformity, to the complete and hopeless mangling usually produced by car wheels.

When a fracture is compounded by the bone end puncturing or lacerating the soft tissues and skin, usually a fairly clean or absolutely clean wound results. Here the baneful effects of hasty action looking to diagnosis or treatment are most apparent. If hemorrhage is of serious nature a tourniquet should be applied until operative steps can be taken to tie the vessel. By no means should the external wound be packed with gauze or other material. If the blood-clot, which soon forms, is touched by a dirty finger, probe or dressing, it is rendered a focus of disease and a menace to the healing process which it otherwise would conserve, as Schede has so well shown. A blood-clot is nature's compress, and it is far safer to carry a patient miles with no other dressing than to infect this clot. In Thornbury's translation of Schimmelbusch's Aseptische Wundbehandlung, it is stated that in half an hour only sixty to seventy bacteria gravitate from the air upon a surface a square decimeter in extent from the germ-laden air of Von Bergman's crowded clinic room, while water from the Spree river carries 37,000 bacteria to the cubic centimeter, and if freely used as an irrigating fluid, unboiled, would bring 37,000,000 bacteria into contact with the wounded surfaces. The number of bacteria on pus-stained dressings, the finger or a probe may not equal this number, but the pathogenic pyogenic bacteria would be present in undue proportion rather than the nonpathogenic germs of putrefaction.

In these cases, then, reduce deformity and immobilise the injured parts for transportation if the patient must be carried some distance. Before doing this, cleanse the wound neighborhood with an antiseptic solution. A 50 per cent. solution of the fifteen volume peroxide of hydrogen is an excellent ready antiseptic. The wound itself should be irrigated superficially with this solution, avoiding disturbing the deeper clot. An aseptic occlusion compress can now be applied and the patient moved home or to such operating place as circumstances require. If the nature of the injury and the appearance of the parts preclude serious infection of the wound, a second more thorough local cleansing and irrigation may now be made without anesthesia. A permanent aseptic occlusion bandage is next applied, and outside this a plaster-of-Paris cast, Volkman's splint, or such dressing as is indicated to reduce deformity, immobilisation being secured. If the condition of the wound is at all in doubt, especially as regards its deeper pockets, anesthetise the patient, enlarge the opening if necessary, thoroughly flush and clear out foreign particles and fragments of bone if stripped from the periosteum and again close the wound by catgut suture, leaving a few strands of catgut for drainage. The occlusion and fixation dressing may be applied as before. Bone fragments should be wired only when deformity cannot otherwise be reduced or immobilisation secured. When infected fractures require frequent change of dressing, wiring is also indicated.

I have noted twelve cases of compound fracture treated as just described. A few of the cases I am able to bring before you. Two of the number I brought before the section last January, one a compound fracture of the humerus and ulna opening into the elbow-joint, and the other a compound fracture of the proximal phalanx of the first finger opening into the metacarpo-phalangeal joint. In both, primary healing of the wound in the soft parts was followed by uninterrupted bony repair and good motion in the joint. Four of the number were compound fractures of the skull, and repair was secured without suppuration. One of these cases is worthy of special report:

Case I.—James Highland, aged 28, entered the Emergency hospital July 9, 1895, after being struck over the left eye by an anchor, while launching a boat. On examination, the skin and occipito-frontalis were found crushed and lacerated and the frontal bone, just over the superciliary ridge, was broken and depressed. Trephining, I removed the bone covering an area of nearly two square inches, exposing the dura mater and opening into the frontal sinus. Trimming the skin edges and opposing them with catgut sutures, and draining with catgut strands, no suppuration occurred, although a sero-sanguineous discharge continued for a week.

I might also report a case of simple fracture of the patella converted into a compound fracture by my opening the joint to wire the fragments. This case I also reported last January, and the healing was typical of what should occur in traumatic compound fractures when bacterial invasion is successfully prevented. Of the four remaining cases two were treated by aseptic occlusion. One case occurred in July, 1895, during my service at the Erie County hospital:

Case II.—A young man, aged 18, was injured by a mowing machine, sustaining a compound fracture of the fibula, an inch above the ankle. Dr. William House, of the house staff, applied an aseptic occlusion dressing, after local cleansing, and the case made a good recovery.

The second case occurred May 29, 1888:

Case III.—George H., aged 12, fell from a wagon, suffering a compound fracture of the right tibia and, probably, simple fracture of the fibula, at the middle of the leg. The primary dressing lay one week and on removal the external wound was closed. The irrigation in this case was superficial, only entering the small external wound and no effort was made to find whether the fibular fracture was also compound. The sublimated irrigating fluid came away without dirt, simply discolored by blood. The deformity and history pointed to protrusion of the upper fragment of the tibia as the cause of the external wound.

The remaining four cases illustrate antiseptic occlusion; two are reported.

Case IV.—Joseph L., aged 40, was kicked by a horse, May 24, 1895, resulting in compound fracture of the right tibia at the junction of the middle and lower third. He was treated by Dr. E. M. Dooley, on his reception at the Emergency hospital, and the wound thoroughly cleansed, irrigated and covered by an antiseptic dressing and a plaster cast. There was slight overriding of the upper fragment and I found him to have marked phosphaturia, union being delayed, but no suppuration developed. He was discharged June 28, 1895.

Case V.—Leon J., aged 26, was also an Emergency hospital case. He is a motorman and on June 2, 1895, he was crushed between trolley cars while adjusting a trolley. On examination I found a compound fracture of the left femur at the junction of the upper and middle third, with laceration and contusion of muscle. Enlarging the skin wound, under ether, I removed pieces of pulpified muscle, bone fragments and clots, using afterward a solution of peroxide of hydrogen to irrigate the wound thoroughly, followed by a solution of corrosive sublimate 1-4000. I then closed the external wound with catgut suture, applied sublimated compresses and a plaster cast to the thigh, put the patient to bed and put the extremity in a Volkman splint with fourteen pounds extension. His temperature reached 101° F. on the third day and then became normal. His plaster cast was not removed for six weeks, but a window was cut in it, over the site of fracture, as oozing sero-sanguineous fluid in the first twenty-four hours was enough to stain through dressing and cast. While walking on the street on July 27th, eight weeks after the original injury, this patient slipped on a wet sidewalk and fell, refracturing the femur at the site of the callus, and was sent back to bed with a plaster-of-Paris dressing for three weeks, when firm, bony union was attained.

When asepsis and antisepsis fail and compound fractures become infected, the main feature is to contend with sepsis in the most effective way until granulation tissue can form and interpose a barrier to the absorption of toxic products by lymphatics and veins. A compound fracture of the humerus in the upper third, in May, 1890, at the Emergency, caused a temperature ranging between 104 and 105 for a week, and it was debated whether amputation of the arm at the shoulder would not offer the patient the best chance for life. Thorough drainage triumphed, but he had lymphangitis, phlebitis and secondary abscesses in the axilla.

In these cases, the key to success is to provide adequate channels for the escape of the toxic products of suppuration. In all cases of compound fracture, when making the first examination under anesthesia, the wound should be digitally explored, with the view of making counter openings later if infection and suppuration make them necessary.

I have noted six infected cases of compound fracture, not counting such injuries of the metacarpals, metatarsals and phalanges. The majority were the result of railroad injury, and the effort was made to prevent septic infection unsuccessfully. In one case the humerus was broken, two were of ulna and radius, one of the tibia, one of the tibia and fibula, and one of the femur. Two deserve special report. In the compound fracture of the femur there was comminution in the lower third. The fragments were removed and the wound drained and packed with iodoform gauze, to check hemorrhage. Suppuration did not appear until ten days later, when gangrene of the leg was well advanced, necessitating amputation in the middle third of the thigh. On examination, thrombosis of the femoral and popliteal artery was found, the clot blocking the lumen of the vessel for five inches. The second case is of interest because spectators of the accident and the patient himself insist that a car wheel ran over the leg above the ankle. As a rule such injuries require amputation. The exception in this instance may be held to prove the rule. He sustained a compound comminuted fracture of the tibia in the lower third, with moderate injury to the soft parts. Dr. E. M. Dooley called me to see the patient five days after the accident and we removed a large fragment of the tibia and made thorough drainage. The result was a useful leg, and the man is now a brakeman on the railroad.

Compound fractures due to gunshot injury are usually considered as a class by themselves. I have had no experience with such injuries, but it seems to me that they should be amenable to treatment according to the principles laid down. Recent investigations go to show that bullet tracks are usually clean, and the modern rifle ball has so high a velocity that perforation and not crushing or splintering will result when it strikes a bone. It is beyond question true that aseptic handling and sterilised dressings for gunshot wounds are giving incomparably better results than the old methods of probing and removing the missile.

It may be argued that compound fractures of the skull should also be considered as a class, *sui generis*. True, the healing process is less complicated and much shorter, as bony repair does not involve the repair of a broken shaft as in the long bones. But, while conservative surgery may classify compound fractures according to the difficulty of successful treatment, as I have done in this paper, it must not differentiate compound fractures of the skull and of the long bones. A compound fracture of the skull, due to the blow of a bludgeon, is to be treated on the same principles as a compound fracture of the humerus, due to a crush between car bumpers. It is fortunate that the skull fracture will heal as kindly as it does, when antiseptically treated, for the sequelæ of septic infection of skull injury are more apt to be fatal than the sequelæ of septic infection in the broken arm.

When the surgeon comes to regard an osteotomy, of the tibia for instance, as analogous to a compound fracture of the same bone caused by the kick of a horse, differing only in the force exerted and the method of its application, he will surely know that the osteotomy is less likely to suppurate, because asepsis and antisepsis have been observed, and he will be prepared to treat all compound fractures that offer any hope to conservative work on the principle of converting them into simple fractures. We cannot as yet examine a wound with the eye of an oil immersion lens in the effort to search out and destroy pathogenic bacteria. When we can do so, the present statistics after treatment of compound fractures can be as much improved as we have improved on the work of the old surgeons, whose principles of treatment are laid down in the gloomy pages of the chapter on prognosis and treatment of compound fracture in the edition of 1880 of Billroth's Surgical Pathology.

771 Ellicott Street.

---

The stringency of the vaccination laws is in England the subject of noisy agitation on the part of ignorant demagogues (*British Medical Journal*); but what would some persons say to the interference with the rights of the individual which is maintained in Norway and Sweden? In these countries so impressed is the legislature not only with the advantages, but with the public duty of vaccination, that before a couple can be legally married certificates must be produced showing that both the bride and bridegroom have been satisfactorily vaccinated.—*Coll. and Clin. Record.*

